# Antibiotic Chemoprophylaxis for Leptospirosis: Previous Shortcomings and Future Needs

**DOI:** 10.3390/tropicalmed9070148

**Published:** 2024-07-02

**Authors:** Kyle Petersen, Ashley Maranich

**Affiliations:** 1 Department of Preventive Medicine, Uniformed Services University of the Health Sciences Bethesda, Bethesda, MD 20814, USA; 2 Department of Pediatrics, Uniformed Services University of the Health Sciences Bethesda, Bethesda, MD 20814, USA; ashley.maranich@usuhs.edu

**Keywords:** leptospirosis, antibiotic, chemoprophylaxis, prevention, human

## Abstract

Leptospirosis is a neglected tropical disease that remains potentially life threatening and hard to diagnose. Climate change combined with overlapping reservoir and human habitats will likely lead to increasing incidence, outbreaks, and mortality in the future. Preventative vaccines are either of limited scope and availability, or under development. Antibiotic chemoprophylaxis for prevention has been the subject of numerous clinical trials. However, despite 40 years of effort, clinical trials to better define protective efficacy, dosing, and the preferred medication are of poor quality and offer limited evidence. We reviewed the literature and offer critiques of the existing trials as well as potential areas for future exploration that may better define the epidemiology and yield a better evidence base for both travel medicine and public health efforts.

## 1. Introduction

Leptospirosis is a bacterial infection caused by species of the spirochete genus *Leptospira*. Although endemic worldwide, incidence is higher in the tropics [1]; the exact epidemiologic data are imprecise but modeling estimates there are 350–500,000 cases worldwide annually and close to 60,000 deaths [2]. By converting Disability Adjusted Life Years to a monetary value based on Gross Domestic Product, a more recent estimate put the lost productivity cost of leptospirosis infection at USD 11.6 billion to USD 52.3 billion with a substantial burden occurring in the Asia-Pacific region [3]. Leptospires are zoonotic and persist in the environment in the proximal renal tubules of mammalian reservoirs, most commonly the brown rat and livestock, as well as in water contaminated by urine of these reservoirs, making leptospirosis a threat in both urban and rural settings [4]. As climatic changes bring more rainfall and tropical storm events, it can only be expected that the risk of leptospirosis exposure will continue to grow across the globe [5]. Certain populations are at higher risk for acquiring leptospirosis. This includes people participating in recreational water sports (particularly when these activities lead to skin injuries, head immersion, or swallowing contaminated water), those exposed to flooding events, and agricultural workers especially of rice and sugarcane crops [6,7,8,9,10,11,12,13,14,15,16,17,18,19,20,21,22]. Additionally, military members on deployments and during training exercises overseas are also at high risk due to the large amount of time spent in field conditions where frequent exposure to soil and water is inevitable. An environmental epidemiologic study from a multi-national military exercise conducted across Thailand in 2018 showed 1.2% of trapped rodents and 30% of environmental samples from the exercise area were positive for Leptospira [23]. Three serosurveys of US service members from Korea, Japan and Honduras showed a 0.9–7.3% seroconversion rate following military deployments to these countries [24,25,26] and a similar 6% seroconversion rate in Mongolian troops deployed to Sudan [27]. Outbreaks show a much higher attack rate ranging in the 20–30% range, but as high as 42% in some units [28,29]. At least seven nations have reported outbreaks in their troops [30]. 

Clinically, many patients are mildly ill or asymptomatic; however, high level (>10^4^ cfu/mL) bacteremia can lead to high levels of inflammatory cytokines such as IL-6, IL-10 and TNF-α. This can result in sepsis or organ failure of the liver, kidneys, or lungs, with the latter having the most severe outcomes. Data on outcomes of pulmonary hemorrhage from leptospirosis have reached as high as 41–56% mortality [9,31,32,33]. Symptoms of leptospirosis are non-specific, with considerable overlap with many other common tropical infections, and confirmatory diagnosis is challenging; PCR is often not available in low- or middle-income countries, culture is technically difficult, and acute serology has a lower sensitivity and specificity than the gold standard Microscopic Agglutination Test, which requires paired sera over time.

Given the challenges with diagnosis, both in clinical recognition and confirmatory testing, it would be ideal to identify high risk patients as candidates for preventive measures. Vaccines for leptospirosis have thus far had limited serovar protection and limited commercial availability [34], so chemoprophylaxis of high-risk populations with antibiotics is currently the best option to prevent severe illness until a universal vaccine is available. A number of clinical trials have been performed for antibiotic prophylaxis of leptospirosis; this review will seek to evaluate their strengths and weaknesses and identify gaps in research that might be targets of future clinical efforts. 

## 2. Medication Related Shortcomings

Only eight studies of antibiotic chemoprophylaxis have been performed since 1984 [35,36,37,38,39,40,41,42]. A summary is included in Table 1. 

The initial study of antibiotic chemoprophylaxis to prevent leptospirosis utilized weekly doxycycline [35]; six other studies have replicated that drug and dosing strategy except for one that also utilized a comparator arm of weekly azithromycin [38] and one that examined daily penicillin [39]. Unfortunately, the initial doxycycline dosing was not based on in vitro effect, small animal models, or pharmacokinetics of doxycycline; the authors chose 200 mg weekly as it was “effective in the prevention of scrub typhus a rickettsial disease with an incubation period similar to leptospirosis” [35]. Doxycycline reaches peak levels in 3–4 h and has a half-life of 12–16 h. Clearly then, a single weekly dose is not killing leptospires for the entire week. More likely with ongoing exposure, leptospires are eradicated with the initial dose, re-infect, and are killed again with the next dose during their prolonged growth phase (5–14 days to clinical symptoms in humans, 6–12 weeks in culture) [36], but there are no studies to confirm this hypothesis. Indeed, 42% of doxycycline recipients in one trial developed leptospirosis [41] and, in an outbreak investigation, taking doxycycline was shown to not be effective in prevention (absolute risk reduction of 0.9 to 3.2%) [28]. Likewise, this weekly dose also failed in scrub typhus, the very disease from which this strategy was adopted [43]. Doxycycline 100 mg daily prophylaxis is a commonly employed strategy for malaria prevention and has good clinical evidence for this usage with a much better steady state than weekly dosing [44,45]. It is often cited by travel medicine experts as being additionally protective against leptospirosis, rickettsia, and plague. We could not find any trials of this dosing for leptospirosis prevention, nor for the other diseases cited.

In addition to dosing strategies, only a few studies addressed adherence to ensure ongoing protection. The daily penicillin study found dosing Penicillin VK 5000 mg twice daily resulted in non-adherence in 47% of subjects after a pill count was conducted [39], which may indicate penicillin is not an ideal drug due to dosing and pill burden complexity, with weekly prophylaxis being preferred due to its simplicity and low pill burden. Three studies utilized directly observed therapy (DOT) [35,40,42] and one DOT for half of the doses [36], with the study team reminding the patient during DOT to take the second dose that night so adherence was likely excellent. Adherence was not accounted for in the other three studies, which makes assessing the protective benefit of prophylaxis difficult. 

Timing of therapy is another problem we noted. Two studies used a single dose at onset of a flooding event: one in the first two days after the flood [40] and the other at day 5–7 after the flood [42], with the former showing no effect and the latter showing 77% protection against seroconversion but no protection against symptomatic infection. This suggests that delaying single dose post-exposure prophylaxis (PEP) for several days may be beneficial in preventing infection. We could not find an effect based on duration of therapy, but the lack of effect in the two single dose trials and protective effect seen in several of the longer weekly studies suggests more than 1 week of prevention as PEP may increase protection.

An early meta-analysis of the first three trials concluded that antibiotics had increased odds of nausea and vomiting with an unclear benefit in reducing seroconversion or clinical consequences of leptospirosis infection and that clinicians should carefully consider their population and whether to use PEP or PrEP [46]. With the benefit of more studies and data since that seminal report, our analysis revealed most studies show medications are well tolerated. Furthermore, in one trial, there were three fatalities among placebo recipients in a high-risk population. Our opinion is that clinicians currently considering prescribing PrEP, especially in light of this mortality information, should consider the risk/benefit balance may be shifting more to favor the benefits with less concerns for side effects of chemoprophylaxis in high-risk populations [36]. In regard to side effects, vomiting occurred in 4.6% of participants in the initial doxycycline trial [35], but that was because the study team gave the last dose on an empty stomach for logistical purposes; the other doses were well tolerated. Nausea comprised 1% of all participants in Thailand (not significant) [42], whereas heartburn or epigastric pain represented 2% and 6% of doxycycline and azithromycin participants in another trial, with only 1% discontinuing medications in both arms [38]. Doxycycline photosensitivity occurred in only one trial in only 4% of subjects [38]. Adverse effects occurred in 1.07% of subjects in one study [37] and were described as “rare and mild” (data not presented) in another study [36]. 

## 3. Diagnostic Shortcomings

Diagnosis of leptospirosis is a challenge; culture is slow and technically hard, as is paired serology using microagglutination testing (MAT). Serology, especially non-quantified assays, will be helpful in finding asymptomatic seroconversion, but can remain positive for a 2–12 years [47], complicating diagnosis in persons with previous exposures. Molecular techniques are emerging but have not been utilized in any trials thus far. Reviewing the methods, we found culture [35] and IgM Immunoassay [40] utilized once each, MAT twice [36,39] and immunofluorescence combined with MAT once [42]. ELISA IgG and IgM (for symptomatic cases) were used once [38] and unspecified ELISA was used once [41]. The methods were not described in one trial [37] and the sensitivity and specificity of the assays is not described in any trial. Because of the widely different methods used for diagnosis, it makes comparing or even combining results for larger analysis very difficult. It is telling that the only trial that found statistically significant protection from prophylaxis utilized culture; however, culture is unlikely to be used in future trials moving forward. In the 4–5 trials that used serology, another challenge was present, that of allowing an adequate amount of time to detect asymptomatic cases. Follow-up serology was performed in a range from 10 days to 12 weeks. One trial performed the MAT only 10 days apart and only for symptomatic cases and probably failed to diagnose two cases, which we classified as probable [39], while another performed it at 2–3 weeks [42], which might have inadequately captured seroconversion, which can sometimes not occur until after three weeks. 

The impact of pre-existing IgG antibodies remains unclear, but one study had 54% seropositivity at baseline [36]. This makes it harder to diagnose both cases and seroconversion, and may provide partial or complete protection against infection. If this is disqualifying for enrollment, it may lead to limited and underpowered studies in highly endemic areas or a need to focus on limited populations such as young children. Performing an epidemiologic serosurvey in an area prior to a prophylaxis trial would be very helpful in assessing baseline rates of exposure. 

## 4. Statistical Shortcomings

There are numerous shortcomings around the statistical analysis of many of these trials. One of the biggest is the outcome chosen. Prevention of death is a frequently used outcome in clinical trials. However, among the eight trials, there were only three deaths reported in one trial [36], which makes this a hard outcome to study as it would require an enormous number of participants to reach significance. Many trials chose to focus on the rare outcome of clinical infection. Only one was able to show statistically significant findings using this outcome [35]; in addition, even combining trials into a meta-analysis in order to increase statistical power failed to show significance for this outcome [46,48,49]. Using asymptomatic seroconversion is an endpoint that is much easier to study, but it asks the question about clinical relevance because preventing asymptomatic seroconversion may not be an important outcome. 

Another issue is lack of statistical power. One trial [39] was not analyzable at all due to the small number of outcomes, and a second failed to reach statistical significance [40]; another trial enrolled the correct number of subjects to find an effect, but did not calculate loss to follow up and became underpowered in the final analysis [38]. This was especially disappointing as it was the only trial to study azithromycin. 

Finally, we come to randomization. One study used a gold standard diagnostic and had adequate power to show protection against seroconversion, but unfortunately the study did not randomize participants and ended up with 619 participants in the doxycycline arm and only 44 in the no medication arm, making it horribly unbalanced [42]. 

## 5. Discussion

Despite 40 years and multiple studies, we still do not have a clear answer about the effectiveness of prophylaxis against leptospirosis. The summations of the latest Cochrane report are quite damning. “We do not know if antibiotics versus placebo or another antibiotic has little or have no effect on all-cause mortality or leptospirosis infection because the certainty of evidence is low or very low. We do not know if antibiotics versus placebo may increase the overall risk of nonserious adverse events because of very low-certainty evidence.” [49]. So, what can investigators do to improve the quality of these studies and ensure better data for the next study?

In terms of medications, trials should choose medicines based on ease of dosing and minimalization of side effects and consider things like current practice in travel medicine to guide these decisions. The high pill count and twice daily dosing used in the penicillin trial and its subsequent high lack of adherence give a clear signal on what is not best practice and demonstrate that a low pill count, and easy dosing schedule is mandatory. Despite its flaws, clearly once weekly 200 mg Doxycycline should remain as a comparator in clinical trials given the strong protective efficacy and trial design that provided the initial evidence of protection. We feel ensuring Directly Observed Therapy in all future trials using this dosing schedule is crucial due to the pharmacokinetic information mentioned previously. In addition, due to its widespread use as an antimalarial, clinicians designing trials should consider 100 mg daily dosing as a novel chemoprophylaxis trial. Newer agents may also be superior to Doxycycline. Omadacycline is a recently approved tetracycline with better pharmacokinetics and could be given 300 mg daily or weekly [50]. Azithromycin clearly warrants more study given its weekly dose and long half-life. In the one trial, it had lower rates of seroconversion, disease and side effects than Doxycycline; these were not significant, however, due to a lack of statistical power [38,49]. In addition, all trial medications should be administered with food to reduce side effects.

In terms of diagnostics, it appears that culture is technically difficult, of limited availability and unlikely to be used in future trials. MAT testing is still fairly available at a number of reference labs and might be considered the current gold standard because it provides serovar data and generally has better specificity than serology and a quantifiable titer. If MAT is not available locally, researchers can always use a serologic method and confirm with MAT at a reference lab, which would strengthen the diagnosis and capture more cases and seroconversions. More important is the use of PCR, which has not been utilized as a diagnostic in any clinical trial of chemoprophylaxis thus far. Pairing PCR with serology seems like an ideal strategy to capture both subjects who present both early and late in infection and newer technologies like LAMP provide a cost-effective and field portable strategy to researchers. 

Finally, clinical trials can be improved by insisting on proven methods to design the study such as prospective trials, randomization of medications, power calculations to assess number needed to show significance and overenrolling to ensure significance in the likely event of loss to follow up. Given the extremely low numbers of clinical cases outside of one or two trials, consideration should be given to developing a leptospirosis human challenge model. This methodology has been successfully developed to provide good clinical trial data for both a virus (dengue) [51] and two parasites (schistosomiasis, malaria) [52,53]. 

## 6. Conclusions

Multiple trials of antibiotic chemoprophylaxis for leptospirosis have hinted at a protective effect, yet all but one are poorly designed or inadequately powered. The low rates of adverse effects coupled with the high protective efficacy in the first trial and apparent protection against mortality and hospitalization in subsequent trials appear to indicate that antibiotic chemoprophylaxis benefits outweigh risk. One or two well-designed trials using randomization, directly observed therapy, thoughtful drug dosing, combination lab testing to increase cases and seroconversions coupled with adequate time for seroconversion, and better statistical planning in the trial design phase should provide additional evidence as to the protective effect of antibiotics and allow for better pre-travel counseling and public health response during flooding emergencies in the future.

## Figures and Tables

**Table 1:** Summary of studies on antimicrobial prophylaxis for leptospirosis.

Reference	Year	Pre or Post Exposure	Antimicrobial(s) Studied	Country	Weaknesses	Strengths
[38]	2018	Pre	Azithromycin 500 mg weekly, doxycycline 200 mg weekly × 12 weeks	Iran	− Enrollment goals did not consider loss to follow up − No protective efficacy noted against clinical illness for any antibiotic (protection only for seroconversion) − Used ELISA IgG as a diagnostic with confirmatory IgM. MAT not used − Only 13 clinical cases	− Significantly less seroconversions in Azithromycin group vs. placebo − Only study of Azithromycin − Fewer seroconversions in Doxy vs. placebo (trend towards significance) − Long time to follow up (adequate time for maximum seroconversion) − Adverse events rare, mostly photosensitivity (8%) only 1% stopped prophylaxis
[39]	2008	Pre	Penicillin VK 500 mg bid × 1 month	Sri Lanka	− 47% non-adherence − Only 5 clinical cases (3 MAT confirmed) − Underpowered to show significance	− Only study of Penicillin − Used MAT as diagnostic
[36]	2000	Pre	Doxycycline 200 mg weekly × 12 weeks	India	− 55% had antibody at baseline in both arms − No difference in seroconversions between Doxycycline and placebo	− Protective efficacy against symptomatic infection significant − Only trial to show mortality benefit − Used MAT as diagnostic
[37]	2012	Pre	Doxycycline 200 mg weekly × 5 weeks	India	− Case count data not available − Diagnostic method not detailed − Incidence, not attack rate, presented	− Incident infection was lower in Doxycycline vs. in controls (0% vs. 7.29%) − Randomized controlled trial
[35]	1984	Pre	Doxycycline 200 mg weekly × 3 weeks	Panama	− 4.6% AEs (vomiting) due to last dose given on empty stomach − 13% seropositive at enrollment − Young healthy homogenous military population − Dosing based on different pathogen	− Only study to use culture for diagnosis − Culture was paired with MAT for diagnosis − Attack rate was significantly lower in doxycycline group vs. placebo (4.5 vs. 0.2%) − Doxycycline provided 95% protective efficacy overall
[41]	2010	Post	Doxycycline 200 mg “weekly” (duration not defined)	India	− Protective Odds Ratio for doxycycline in univariate analysis was lost in multivariate analysis − Only ELISA IgM test used, no MAT	− Age- and sex-matched controls − Multivariate analysis
[42]	2014	Post	Doxycycline 200 mg single dose	Thailand	− Non-randomized trial, study arms unbalanced (619 Doxy 44 no doxy) − Single dose may not be sufficient protection − Protective efficacy not significant for clinical illness (only 6 clinical cases) − Follow-up serology only 2–3 weeks after initial may not have allowed for 100% seroconversion	− Doxycycline significantly protective against leptospirosis seroconversion − MAT testing used to confirm IgG − Only trial to study hospitalizations − Only study to show skin injury effect on seroconversion and illness
[40]	1998	Post	Doxycycline 200 mg single dose	Brazil	− Study underpowered (82 subjects); no significant doxycycline effect on illness or seroconversion − Single dose may not be sufficient protection	− 45 days between paired serum adequate to allow seroconversion

## Data Availability

Not applicable.
